# Transcriptional Regulation of Cellulose Biosynthesis during the Early Phase of Nitrogen Deprivation in *Nannochloropsis salina*

**DOI:** 10.1038/s41598-017-05684-4

**Published:** 2017-07-13

**Authors:** Seok Won Jeong, Seung Won Nam, Kwon HwangBo, Won Joong Jeong, Byeong-ryool Jeong, Yong Keun Chang, Youn-Il Park

**Affiliations:** 1 Department of Biological Sciences, Chungnam National University, Daejeon, 34134 Korea; 2 Bioresources Culture Collection Division, Nakdonggang National Institute of Biological Resources, Sangju, 37242 Korea; 3 Korea Research Institute of Bioscience and Biotechnology, Daejeon, 34141 Korea; 4 Department of Chemical and Biomolecular Engineering, Korea Advanced Institute of Science and Technology, Daejeon, 34141 Korea; 5 Advanced Biomass R&D Center (ABC), Korea Advanced Institute of Science and Technology, Daejeon, 34141 Korea

## Abstract

Microalgal photosynthesis provides energy and carbon-containing precursors for the biosynthesis of storage carbohydrates such as starch, chrysolaminarin, lipids, and cell wall components. Under mild nitrogen deficiency (N−), some *Nannochloropsis* species accumulate lipid by augmenting cytosolic fatty acid biosynthesis with a temporary increase in laminarin. Accordingly, biosynthesis of the cellulose-rich cell wall should change in response to N− stress because this biosynthetic pathway begins with utilisation of the hexose phosphate pool supplied from photosynthesis. However, few studies have characterised microalgal cell wall metabolism, including oleaginous *Nannochloropsis* sp. microalgae subjected to nitrogen deficiency. Here, we investigated N-induced changes in cellulose biosynthesis in *N. salina*. We observed that N− induced cell wall thickening, concurrently increased the transcript levels of genes coding for UDPG pyrophosphorylase and cellulose synthases, and increased cellulose content. *Nannochloropsis salina* cells with thickened cell wall were more susceptible to mechanical stress such as bead-beating and sonication, implicating cellulose metabolism as a potential target for cost-effective microalgal cell disruption.

## Introduction

Unicellular and nonmotile *Nannochloropsis* microalgae synthesise neutral triacylglycerol (TAG) lipids at levels that can reach up to 60% of the dry cell weight (DW), and TAGs can be transesterified and used as feedstock for biodiesel conversion^1^. *Nannochloropsis* also contain abundant polyunsaturated fatty acids (PUFAs) such as eicosapentaenoic acid (EPA). EPA is a functional food with beneficial effects such as heart disease prevention and anti-inflammatory activity, and positive effects on brain development and vision^2^. *Nannochloropsis* has emerged as an oleaginous model microalga due to high photosynthetic efficiency and lipid productivity, and advanced biotechnology applications including a well-established genetic toolbox and large-scale outdoor cultivation systems^3–6^.

Algal cell walls contain carbohydrates, proteins, and lipids, which protect the cells from adverse environmental conditions. Although microalgae have potential as feedstock for biofuel production, the cell wall affects every step of the process, including algal growth, harvesting, dewatering, and extraction^7, 8^. Cell wall thickness in *Nannochloropsis* species varies from 63 to 119 nm due to the distinct genetic traits in each strain^9^. Changes in culture conditions, such as reductions in the concentrations of salt, nitrogen, phosphate, and sulphur induce cell wall thickening in *Nannochloropsis*, *Chlorella*, *Chlorococcum*, and *Chlamydomonas* ^9–11^. Algal cell wall properties are important for biotechnological applications. However, few studies^11^ have investigated the effects of cultural stress conditions and nitrogen deficiency (N−) on the biosynthesis of cell wall components and TAG production in the majority of algal species.

Cellulose is the major cell wall polysaccharide in *N. gaditana* (~75%)^8^ and *N. oculata* (~80%)^12^, and it forms the inner layer of the bilayer cell wall. The outer wall contains layers of long-chain aliphatic hydrocarbons such as algaenan^8, 13^. As in other organisms, cellulose biosynthesis in *Nannochloropsis* is initiated by the formation of UDP-glucose (UDP-Glc) from glucose-6 phosphate (Glc-6-P) via UDP-Glc pyrophosphorylase (UPP), followed by cellulose biosynthesis via cellulose synthases (encoded by *CesA* genes) that utilise UDP-Glc and β-1,4-glucan^5, 8^. A survey of publicly available genomic data for *N. salina*, *N. gaditana*, and *N. oceanica* (http://www.bioenergychina.org/fg/d.wang_genomes/) reveals the presence of one *Upp* and four *CesA* genes, but the biosynthetic pathway for algaenan production has not been elucidated.

In this study, we examined the biosynthesis of cellulose and TAGs in the oleaginous microalga *N. salina* CCMP1776 subjected to N−. First, we observed that N− induced parallel increases in cellulose and TAG. Nitrogen deficiency also increased the levels of *Upp* and *CesA* transcripts and concurrently increased the expression of genes involved in cytosolic fatty acid and lipid biosynthesis, including cytosolic fatty acid synthase type 1 (*Fas1*), diglyceride acyltransferase (*Dgat-2*) and ∆12-desaturase (*Fad2*). Consistently, N− increased cellulose content and induced cell wall thickening, which caused cell wall fragility. These properties render the cells more susceptible to mechanical stress disruption, which can improve their performance as feedstock for biofuel reactors.

## Results

### Growth, photosynthesis, and morphology of *Nannochloropsis salina* cells in response to nitrogen deprivation

**Figure 1:**
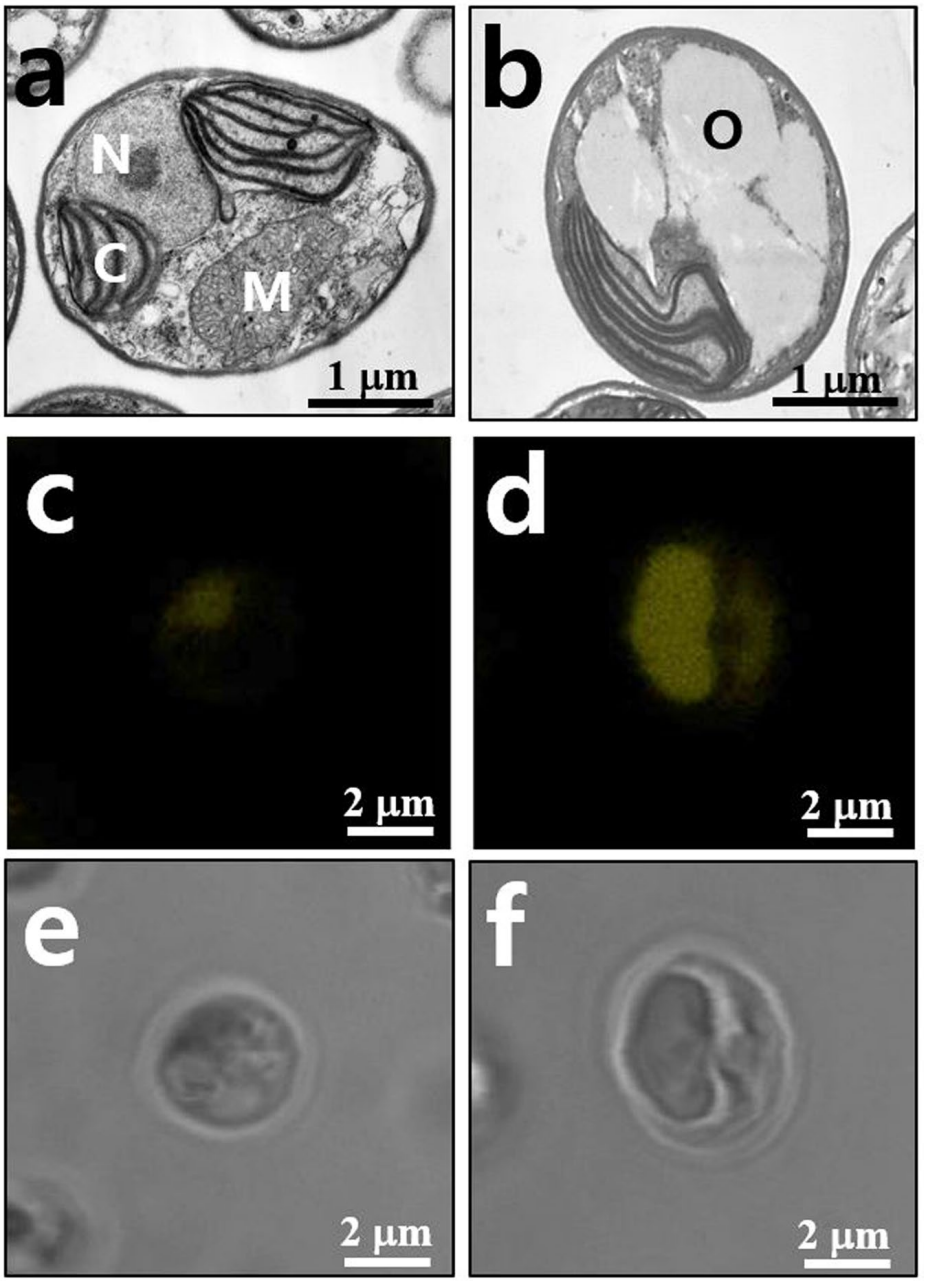
Representative transmission electron microscopy (TEM) (**a**,**b**), Nile Red fluorescence (**c**,**d**), and bright field (**e**,**f**) images of *N. salina* cells grown under N+ and N− conditions for 2 days. Cells were fixed and then subjected to TEM analysis. Organelles are indicated as follows: N, nucleus; M, mitochondria; C, chloroplast; O, putative oil bodies. Oil bodies stained with Nile Red are visible (yellow) inside the cells.

Nitrogen deficiency (﻿N−) is the most effective external factor for triggering TAG production^14, 15^; it causes a series of physiological changes in some *Nannochloropsis* species including growth retardation, enhanced cell size, pigment alterations and lipid accumulation, and it reduces photosynthesis, carbon fixation, and protein synthesis^11, 15, 16, 17^. We were interested in the response of *Nannochloropsis* cell wall metabolism to N−, where photoassimilates are known to be partitioned primarily into the synthesis of storage carbohydrate and lipid. First, we established batch culture conditions where TAGs accumulate in the oil body and photosynthetic electron transport activities remain essentially unchanged. This allowed us to examine photoassimilate partitioning between structural and storage pools in algal cells subjected to N−. Growth curves for *N. salina* cells grown in f/2 medium completely lacking N showed significantly reduced growth rates compared with those for cells transferred to N-containing medium (Supplementary Fig. S1). *N. salina* cells accumulated oil bodies 2 days after transfer to N− medium, which accounted for a major fraction of the cell volume (Fig. 1a *vs*. b), and displayed 83.1% reduction in specific growth rate and 118% increase in cell diameter (Table 1). Under these N− conditions, the cell cultures became yellowish green (Supplementary Fig. S1b) due to strong reductions in Chl *a* (approximately 76%) and carotenoids (Car) (approximately 53%). By contrast, control N+ cultures were green (Supplementary Fig. S1b). The reduction in photosynthetic pigments in response to N− was not caused by decreases in the content of active PSII (Simionato *et al*.^16^) as *in vivo* Chl fluorescence parameters [F_v_/F_m_ and 1−F/F_m_′ (ΦPSII)] were comparable between N+ and N− cells (Table 1). Consistently, chloroplast membranes remained essentially intact, indicating that the thylakoid membrane organisation of *N. salina* cells subjected to N− was very similar to that observed in *N. salina* cells subjected to N+ (Fig. 1a,b).

**Table 1:** Changes in specific growth rate (SGR), cell size, chlorophyll *a* (Chl) and total carotenoid (Car) contents, maximal (F_v_/F_m_), and effective quantum yield of photosystem II (ΦPSII) in darkness and during illumination in *N. salina* cells grown under nitrogen-rich (N+) and nitrogen-depleted (N−) conditions for 2 days.

Sample	SGR (d^−1^)	Cell size (μm)	Chl (fg/cell)	Car (fg/cell)	Car/Chl	F_v_/F_m_	ΦPSII
N+	0.53 ± 0.07	2.73 ± 0.41	96.09 ± 0.49	26.69 ± 0.21	0.28 ± 0.01	0.64 ± 0.02	0.27 ± 0.03
N−	0.09 ± 0.08	3.24 ± 0.45	23.30 ± 0.38	12.54 ± 0.41	0.54 ± 0.01	0.67 ± 0.01	0.29 ± 0.01

Data are expressed as the average of three biological replicates ± SD. Each sample was significantly different from the other (independent two-tailed *t*-test, *p*-value < 0.05). Cell size was measured from TEM images.

### Lipid contents in *Nannochloropsis salina* cells in response to nitrogen deprivation

Photosynthetic activities were comparable in cells subjected to N+ and N− growth conditions. By contrast, lipid metabolism was significantly enhanced under N− growth conditions. First, electron microscopy imaging of N− cells clearly revealed that electron-opaque oil bodies occupied a major fraction of the cell volume (Fig. 1a,b). Second, *N. salina* cells stained with the lipid soluble Nile Red (NR) dye, which detects neutral lipids^18^ in a concentration-proportional manner^19^, exhibited strong yellow fluorescence emission at 580 nm from NR dye incorporated into intracellular oil bodies (Fig. 1c,d). NR fluorescence emission from N− cells was 6.72-fold higher than that from N+ cells (Table 2). Two days of nitrogen deprivation also caused a 2.01-fold increase in the total fatty acid contents (Table 2). FAME analysis of fatty acid composition in nitrogen-deprived cells indicated that palmitic acid (C16:0), palmitoleic acid (C16:1) and oleic acid (C18:1) contents were enhanced by 167%, 119.4% and 272.1%, respectively. However, the contents of PUFAs such as eicosatetraenoic acid (ETA, C20:4) and EPA (C20:5) decreased by 70.1% and 72.0%, respectively, in response to nitrogen deprivation (Table 2). These results were similar to those observed in chronically N-deprived *N. oceanica* ^20^. These combined results suggest that acute responses to N− stress include increases in saturated and monosaturated fatty acid contents and reductions in PUFAs.

**Table 2:** Changes in neutral lipid and total fatty acid (TFA) contents, and fatty acid compositions from *N. salina* cells grown under nitrogen-rich (N+) and nitrogen-depleted (N−) conditions for 2 days.

Sample	Neutral lipid (F_580_)	TFA (g/g DW)	Fatty acid composition (%)				
			Palmitic acid (C16:0)	Palmitoleic acid (C16:1)	Oleic acid (C18:1)	ETA (C20:4ω6)	EPA (C20:5ω3)
N+	9.98 ± 0.91	0.17 ± 0.02	21.18 ± 0.11	33.04 ± 0.61	2.01 ± 0.07	7.70 ± 0.36	16.27 ± 0.58
N−	67.09 ± 2.95	0.34 ± 0.03	35.43 ± 0.21	39.45 ± 0.14	5.47 ± 0.03	2.16 ± 0.14	4.87 ± 0.18

Neutral lipid content was estimated by Nile Red fluorescence amplitude at 580 nm excited at 430 nm. Only significantly different fatty acid species between the two samples are represented. Data are expressed as the average of three biological replicates ± SD. Each sample was significantly different from the other (independent two-tailed *t*-test, *p*-value < 0.05). EPA, eicosapentaenoic acid; ETA, eicosatetraenoic acid; TFA, total fatty acid.

### Transcript levels of genes involved in cytosolic fatty acid and TAG biosynthesis

**Figure 2:**
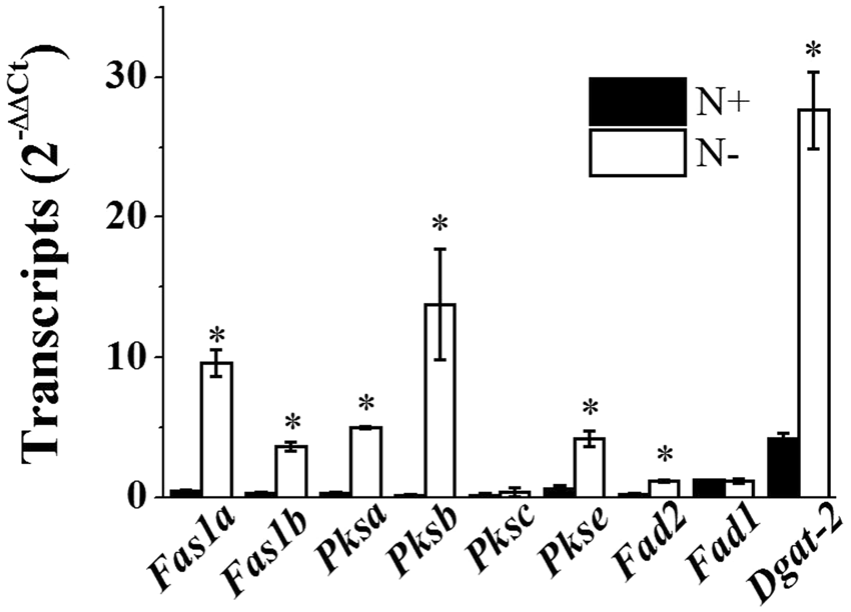
Transcript levels of cytosolic fatty acid and lipid biosynthesis genes in *N. salina* cells grown under N+ and N− conditions for 2 days. Each data point represents the mean ± SE of three biological and technical triplicate culture flasks.

In microalgae, N−-induced lipid accumulation depends primarily on *de novo* fatty acid (FA) biosynthesis and partly on glycerolipid degradation of the thylakoid membrane via the recycling galactolipids-to-TAG route as described previously in *C. reinhardtii* ^21^ and *N. gaditana* ^16^. In *Nannochloropsis*, biosynthesis of FAs and thylakoid membrane lipids occurs at the chloroplast stroma and envelope, respectively, whilst the major phosphoglycerolipids, betaine lipids, and TAGs are synthesised in the endoplasmic reticulum (ER)^5, 22–24^. In N− *Nannochloropsis*, accumulation of TAGs (C16:0 and C18:1) is due to *de novo* FA and TAG biosynthetic pathways rather than thylakoid membrane conversion^25^. In contrast with other microalgae, *Nannochloropsis* harbours putative genes coding for cytosolic *Fas1* or polyketide synthase (*Pks*) in addition to plastid *Fas2* ^5, 26^. FAS is a multi-enzyme complex involved in the synthesis of palmitic acid (C16:0) and stearic acid (C18:0). The *N. salina* genome contains two putative *Fas1* (*Fas1a* and *Fas1b*) and four *Pks* (*Pksa, Pksb, Pksc*, *and Pkse*) genes. Several-fold increases in the transcript levels of these *Fas1*/*Pks*-like genes were observed in N-deprived *N. salina* cells (Fig. 2).

The ∆12 desaturase FAD2, which is encoded by *Fad2* and located in the ER membrane facing the cytosol, desaturates different glycerol-backbones such as phosphatidylcholine and monogalactosyldiacylglycerol with similar efficiencies^27^, and possesses high substrate specificity toward oleic acid (18:1*n*-9) (50% conversion)^28^. ∆5 desaturase, which is encoded by *Fad1*, adds a double bond at the fifth carbon-carbon bond from the carboxylic acid end in FAs such as dihomo-gamma-linoleic acid (DGLA, 20:3*n*-6) and ETA (20:4*n*-3) to produce arachidonic acid (ARA, 20:4*n*-6) and EPA, respectively^29^. Consistently, overexpression of a putative ER-localised FAD1 significantly enhanced the accumulation of both monounsaturated and polyunsaturated fatty acids in the marine diatom *Phaeodactylum tricornutum* ^30^. DGAT catalyses the last step of TAG synthesis from DAG and acyl-CoA^31^, and includes DGAT-1 and DGAT-2^32^. There are 12–13 *Dgat* genes in each *Nannochloropsis* strain (1–2 *Dgat-1* and 11 *Dgat-2*), representing the highest dose among known genomes^5, 33^. Accordingly, considering the significant increases in lipid bodies and some FA species observed, *Dgat-2* and *Fad2* were selected from the genes involved in TAG and FA biosynthesis for measurement of transcript levels (Fig. 1 and Table 1). As expected, N− enhanced the transcript levels of *Fad2* and *Dgat-2* by 6.6- and 5.4-fold, respectively (Fig. 2). Although both EPA and ETA contents declined in response to N−, the *Fad1* transcript level remained unchanged (Fig. 2).

The transcript levels of some genes involved in FA biosynthesis in the chloroplast, such as malonyl-CoA:acyl carrier protein transacylase (*Mct*), 3-ketoacyl-acyl carrier protein reductase 1 (*Kar1*), 3-hydroxyl acyl dehydratase (*Had1*) and branched-chain α-keto acid dehydrogenase subunit E2 (*Bcdh*), remained unchanged or only marginally changed compared with those of cytosolic enzymes (Supplementary Fig. S2). These concurrent increases in transcript levels of genes involved in cytosolic FA and TAG biosynthesis and their contents suggest that oil body accumulation is under transcriptional control in *N. salina* subjected to N deficiency.

### Cell wall thickening in *Nannochloropsis salina* cells in response to N deprivation

**Figure 3:**
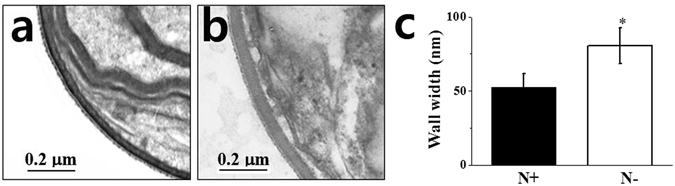
Representative TEM images of cell wall (**a**,**b**) and cell wall width (**c**) of *N. salina* cells grown under N+ and N− conditions for 2 days. In (**c**), the cell walls of a total of 250 individual N+ or N− cells were measured in five separate places.

**Figure 4:**
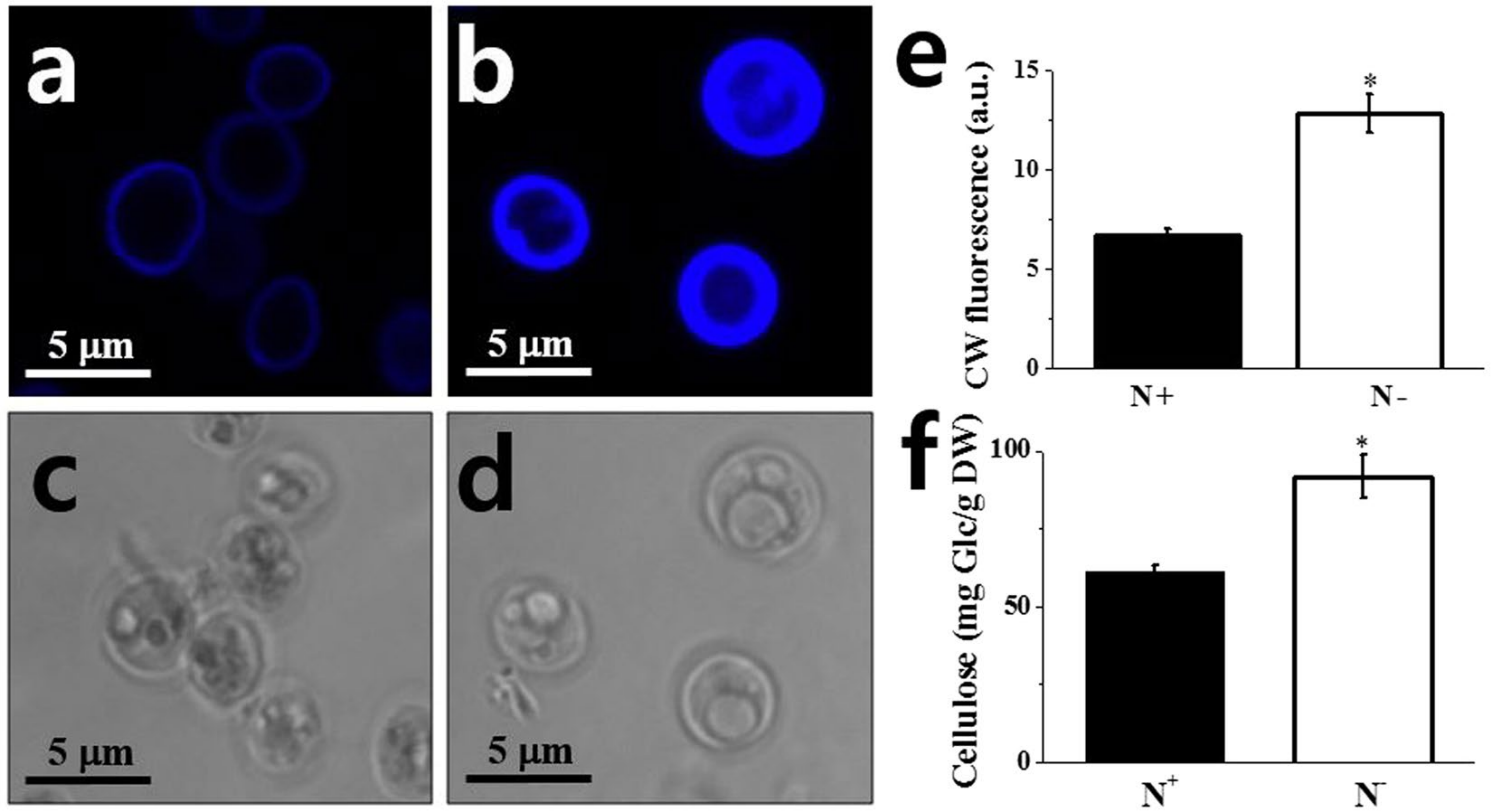
Representative images of calcofluor white (CW) fluorescence (**a**,**b**), and bright field (**c**,**g**), and quantification of CW fluorescence intensity (**e**) and cellulose (**f**) of *N. salina* cells grown under N+ and N− conditions for 2 days. In (**f**), glucose (Glc) released from cell wall extracts by cellulase (1%, w/w) treatment for 24 h were quantified. Each data point represents the mean ± SE of three biological and technical triplicate culture flasks.

The ultrastructural images of *N. salina* cells showed that N-limited cells exhibited a 1.54-fold thicker cell wall than that of N-complete cells due to apparent swelling of the inner cell layer (Fig. 3a–c). To determine cell width increases, we stained cells with calcofluor white (CW), which specifically binds to structures containing cellulose and chitin, and has been used to stain cell walls of both algae and higher plants^34^. As shown in Fig. 4a,b, *N. salina* cells stained with CW emitted ring-shaped fluorescence outside the cells, which was 1.9-fold stronger (Fig. 4e) in N− cells than in N+ cells.

The cell wall mass of *N. salina* as a percentage of DW is currently unknown. Cellulose is the major cell wall polysaccharide in *Nannochloropsis* species, such as *N. gaditana* (~75%)^8^ and *N. oculata* (~80%)^12^ and forms the inner layers of cell walls exhibiting a trilaminar organization. The finding that N deficiency induced CW fluorescence enhancement strongly hints that cell wall thickening is due to an increase in cellulose content. Therefore, we quantified cellulose content in purified cell wall materials. Algal cell walls are usually obtained by mechanical cell breakage with beads, ultrasonification or high pressure homogenization^35^. In the present study, bead beating was adopted for cell breakage and residual proteins and lipids were removed by phenol and chloroform extraction. Cell wall mass after enzymatic removal of laminarin contamination was ~20% of DW, which is *ca*. 10-fold higher than in *N. gaditana* ^8^. The difference in cell wall mass between this study and the study of Scholz *et al*.^8^ may be due to differences in the cell breakage methods used in the two studies. By contrast to the method employed in this study, *N. gaditana* cells were ruptured with a French press followed by sucrose gradient centrifugation to remove other cellular components in the study by Scholz *et al*.^8^. However, this process results in incomplete cell breakage and loss of cell contents, which makes it hard to quantify cell wall mass accurately. The increases we observed in cellulose content under N deficiency (Fig. 4e) was confirmed by quantification of the amount of Glc released from cellulose by cellulase treatment, which showed that Glc content was 1.50-fold higher than that of N+ (Fig. 4f).

**Figure 5:**
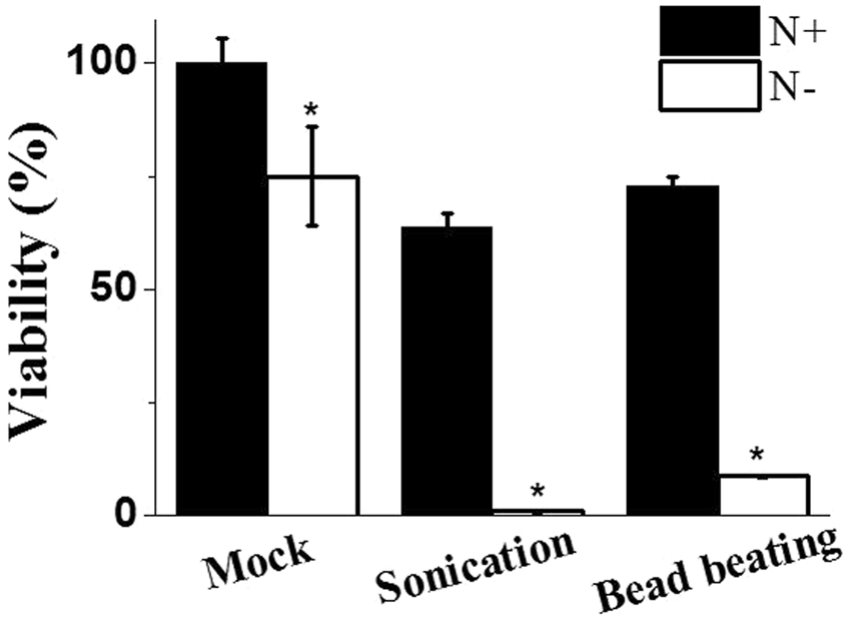
Cell viability after mechanical stress of bead-beating and sonication in *N. salina* cells grown under N+ and N− conditions for 2 days. Each data point represents the mean ± SE of three biological and technical triplicate culture flasks.

Increased cell size would make the cells more vulnerable to sheer forces and the cell walls more susceptible to breakage, even with increased wall thickness, as the tensile strength of an algal cell wall does not necessarily correlate with wall thickness^36^. To test this hypothesis, cells were subjected to two mechanical disruption methods, bead-beating and sonication. In bead-beating, beads are ground against cells, which causes cell lysis. By contrast, cell lysis by sonication is attributed to cavitation, where rapid formation of gas bubbles creates high pressure and temperature^37^. Cell disruption efficiencies were assayed by counting the colony number of viable cells after mechanical stresses instead of by the cell digestion assay, and cell staining for nucleic acids or viability using SYTOX^®^ Green and membrane-permeable Neutral red, as these staining methods were associated with problems such as autofluorescence and algal cell size-dependent incorporation^38^. As shown in Fig. 5, the viability of cells subjected to N deprivation for 2 days decreased by 25% compared with that of control cells. Cells subjected to N deprivation and mechanical stresses such as bead-beating and sonication exhibited significantly reduced viability. This indicates that N− increases cell wall thickness and causes increased susceptibility to mechanical stresses.

### Transcript levels of genes involved in cellulose biosynthesis

**Figure 6:**
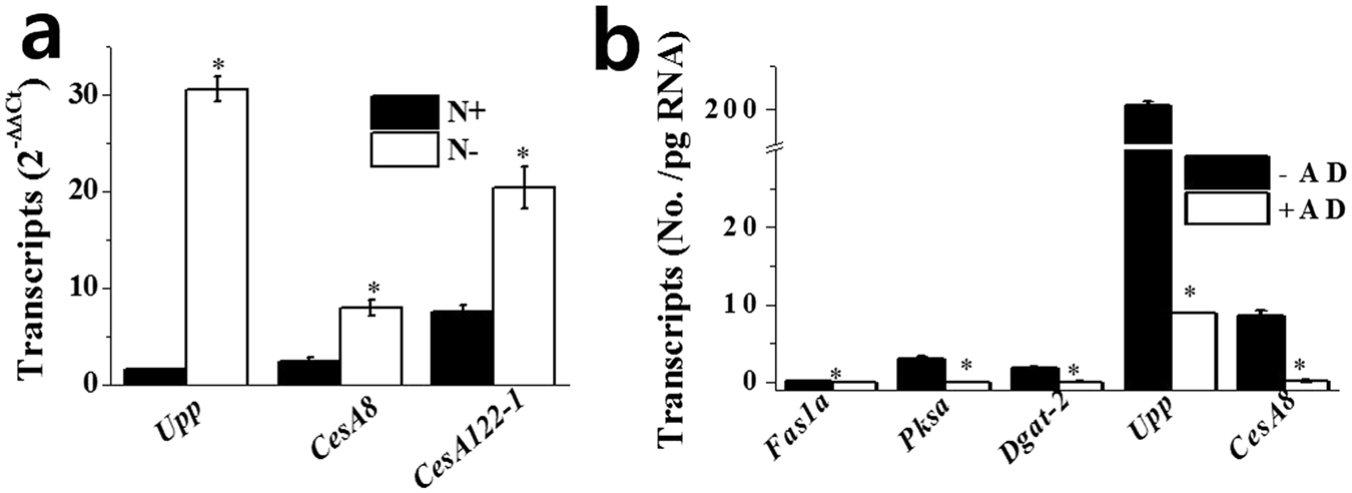
Transcript levels of genes involved in cellulose (*Upp*, *CesA8*, and *CesA122-1*) (**a**) and fatty acid (*Fas1a* and *Pksa*), lipid (*Dgat-2*), and cellulose (*CesA8*) (**b**) biosynthesis in *N. salina* cells grown under N+ and N− conditions for 2 days. In (**b**), transcription inhibitor actinomycin D (AD, 100 μg ml^−1^) was included in N− media for 2 days prior to the onset of N− growth. Absolute copy numbers of the mRNA transcripts (number of copies/ng of total RNA) were determined by qRT-PCR analysis. Each data point represents the mean ± SE of three biological and technical triplicate culture flasks.

Cellulose synthases are essential housekeeping genes for primary cell wall synthesis in higher plants; therefore, *Ces1*, *-3* and *-6* transcripts ubiquitously accumulate to high levels^39^. At the same time, cellulose biosynthesis in higher plants is transcriptionally regulated by hormones such as ethylene and brassinosteroid (BR)^40^. We investigated whether cellulose accumulation in response to N− was correlated with increases in the transcript levels of cellulose biosynthesis genes. The results showed that cell wall thickening induced by N− was positively correlated with enhanced transcript levels of genes involved in cellulose biosynthesis such as *Upp* and *CesA*; the transcript levels of *Upp*, *CesA8*, and *CesA122-1s* were enhanced by 20.1-, 3.3-, and 2.7-fold, respectively (Fig. 6a).

To determine whether N deficiency-induced increases in FAs, TAGs, cellulose contents, and transcript levels of some genes involved in their biosynthesis are transcriptionally regulated, *N. salina* cells were treated with actinomycin D for 48 h to inhibit mRNA transcription before harvesting cell lysates and determining mRNA levels. Actinomycin D blocked increases in NR and CW fluorescence that were induced by N−, resulting in 77.8% and 52.6% of the fluorescence of untreated control cells, respectively (Supplementary Table S1). The levels of genes involved in FA, TAG, and cellulose biosynthesis such as *Fas1a*, *Pksa*, *Dgat-2*, *Upp*, *CesA8*, and *CesA122-1* also were significantly reduced, with levels approximately 1.7–4.0% of those in control cells (Fig. 6b). The transcriptional dependence of increased mRNA levels indicates that increased transcription rates likely cause photoassimilate partitioning into storage and structural polysaccharides under N−.

## Discussion

Cell disruption is an important but expensive step during the extraction of lipids in algal biodiesel production^8^. Cell wall thickness not only affects the carbon budget, ease of downstream oil extraction, and residual biomass composition^7^, but it is also a major barrier to bioengineering^9^. Thus, metabolic engineering combined with varying culture conditions such as light intensity, temperature, salinity, and nutrients is a potential target for developing microalgal-based biofuel production. Nitrogen deficiency induces cell wall thickening in some algae such as *Nannochloropsis* sp., *Chlorella* sp., *Chlorococcum* sp., and *Chlamydomonas* ^10, 11^. In the present study, we tested whether N− caused changes in cell wall biosynthesis and resulted in cell wall thickening in *N. salina*. We selected *N. salina* out of nine *Nannochloropsis* species because it had the highest biomass productivity (0.36 g l^−1^ day^−1^ *vs*. 0.18–0.32 g l^−1^ day^−1^) and the lowest contents of neutral lipids (37.0% *vs*. 41.2–60.4%) and TAGs (23.8% *vs*. 30.9–58.4%)^1^, and it was robust to changes in salinity^11^. High biomass production would be advantageous for commercial applications using two-stage N deprivation cultivation^41^. The low lipid contents in *N. salina*, although still high when compared with other microalgae, could be a potential target for biotechnological improvement of lipid productivity. In the present study, we tested whether N deprivation could dramatically enhance oil production in *N. salina*. Then, under augmented lipid production conditions, we also investigated changes in transcript levels of genes involved in cellulose biosynthesis, as photoassimilate partitioning to structural carbohydrates could be a potential target for genetic manipulation.

Here, we achieved 2-fold increases in total FA contents per cell in response to N−, from 17% to 34% of DW. This increased oil production resulted from remobilisation of chloroplast membrane lipid or degradation of carbohydrate reserves, which accumulated as in chronically N-deprived cells, rather than from photosynthesis, which is the dominant energy source for TAG biosynthesis^16^. Consistently, photosynthetic activities and chloroplast ultrastructure remained unchanged in *N. salina* cells starved for nitrogen for 2 days. The transcript levels of *Fas1*/*Pks*-like genes were strongly upregulated by N− in *N. salina*, which was not observed in the presence of the transcriptional inhibitor actinomycin D. These combined results suggest that transcriptional regulation of FA biosynthesis is important. Nitrogen-induced changes in FA composition (high levels of C16:0 and C18:1 FAs with concomitantly reduced levels of PUFAs such as C20:5) support the proposal that *de novo* synthesised FAs (rather than converted membrane lipids) are directly incorporated into TAGs via the Kennedy pathway^42^. Similarly, in N-stressed *N. gaditana*, enhanced lipid biosynthesis was attributed to the transcriptional activation of cytosolic FA biosynthesis^16, 43, 44^.

Under N− conditions, photosynthesis actively supplies energy and photosynthetic carbon intermediates including glyceraldehyde-3-phosphate, and *N. salina* cell walls were approximately 1.54-fold thicker than controls, which is attributed primarily to cellulose accumulation. Cellulose biosynthesis starts from Glc-6-P, which is in turn generated from triose-P pools. Thus, during the early stage of N−, the photosynthetic assimilate triose-P would channel into the biosynthesis of lipids and cell wall components (Supplementary Fig. S4). This enhanced cellulose biosynthesis is positively correlated with observations of several-fold increases in the transcript levels of *Upp* and *CesA*, which were completely sensitive to the transcriptional inhibitor actinomycin D, again suggesting transcriptional regulation of cell wall biosynthesis similarly as in lipid biosynthesis. Consistently, we observed weaker resistance to mechanical stress in cells with thickened cell walls compared with control cells, strongly suggesting that metabolic perturbations induced by N− could be a method to change the cell wall mechanical properties. Increasing the light intensity^45, 46^ and medium salinity^11, 16, 43^ during culture also enhanced cytosolic TAG accumulation in *N. gaditana*, most likely via transcriptional activation of the FAS1/PKS-like protein Kennedy pathway if the CO_2_ level was not limiting^47–49^. Future work should evaluate whether light intensity and medium salinity, or a combination of these stress factors, also increases cell wall biosynthesis and results in cell wall thickening. Ectopic expression of *Upp* and *CesA* by transient expression systems induced by nitrogen deprivation would be useful for designing *N. salina* cells with a specific cell wall thickness that would enable optimal cell growth but require the least energy for cell disruption. These results suggest that diverting carbon from cell wall synthesis toward lipid production during the biomass production phase is not necessarily beneficial for lipid-based biodiesel production.

The *N. salina* carbon partitioning model (Supplementary Fig. S4) can guide genetic engineering for enhanced TAG productivity, as biosynthesis of storage carbohydrates, cellulose and neutral lipids in microalgae may compete for the same sources of carbon precursors (triose-P) and reducing power. Laminarin is the cytosolic storage carbohydrate in *Nannochloropsis* and accounts for 5–8% of the DW. Among putative β-glucan synthesis enzymes, the transcript levels of phosphoglucomutase (PGM), UDP-sugar pyrophosphorylase (UPP), 1,3-β-glucan synthase (BS), and β-1,3-glucosyltransferase (BGT) were induced (with different induction rates) in *N. oceanica* under N− conditions, whilst those of glucosyl hydrolase (GH), two endoglucanases (EG and EGA), and two β-glucosidases (bGS and bGSG) involved in β-1,3-glucan degradation remained unchanged^25^. Consistently, N deprivation strongly enhanced the transcript levels of *Bs* (2.7-fold) and *Bgt* (13.8-fold) in *N. salina*, which were significantly reduced by actinomycin D treatment (Supplementary Fig. S3). This suggests that β-1,3-glucan may serve as a temporary carbon storage form, and its biosynthesis may be transcriptionally regulated as in *N. oceanica* ^25^. Therefore, knockdown or knockout of these genes (e.g., *Bgt* and *Bs*) should reduce the accumulation of β-1,3-glucan and shunt the carbon flow to competing pathways such as TAG and cellulose biosynthesis. Genes such as *Fas1* and *Fad2* may be promising candidates to divert carbon intermediates into TAG biosynthesis, because they showed remarkable upregulation under N− conditions.

Biosynthesis of *Nannochloropsis* carbon reserves such as mannitol, galactose, and β-1,3-glucans and cellulose starts from the hexose-P pool containing Glc-1P, Glc-6P, and fructose-6P^25^, which are replenished from photosynthetic activities. Concurrent changes in the expression levels of genes observed in the present study implies that enhanced photosynthate utilisation is likely regulated at the transcriptional level by master transcription factor(s) under N deprivation conditions. A recent study reported that basic helix-loop-helix (bHLH) from *N. salina* has a dominant role in activating FA biosynthesis genes^50^, suggesting that these transcription factors may be a potential master switch. Random mutagenesis could be used in the near future to screen for mutants with defective growth and photosynthesis, and to identify the master switch (switches) and regulon (regulons) that mediate the global activation of cellulose, lipid, and storage carbohydrates in response to N−.

N-induced cell wall thickening resulted in fragile cell walls. This result was not consistent with a recent report that N-induced cell wall thickness in *Nannochloropsis* sp., *Chlorella* sp., and *Chlorococcum* sp. did not significantly affect cell susceptibility to mechanical rupture^11^. These differences may be due to differences in the mechanical rupture methods; the present study used sonication and bead-beating, whilst the previous study used a high-pressure homogeniser. Sonication and high-pressure homogenisation share a similar disruption mechanism, which involves cavitation bubbles^37^. Another reason for the differences could be due to differences in the time when the number of intact cells was counted. Yap *et al*.^11^ counted cell numbers immediately after homogenisation, whilst we counted the number of surviving colonies on agar plates after 25 days of growth on f/2 medium. Cell homogenisation changed the cell size distribution curves compared with those before homogenisation, increasing the percentage of cells with small diameters at the expense of the percentage of cells with large diameters^51^. The smaller cells could arise from the loss of cell contents during homogenisation as observed in *Chlorella vulgaris* subjected to sonication-assisted homogenisation^51^. If this is the case, then counting the remaining cells immediately after homogenisation would overestimate the number of intact cells.

The *Nannochloropsis* cell wall component algaenan is very similar to cutan, which occurs in drought-resistant land plants such as *Agave* and *Clivia*, and contains long-chain (~C_30_) alkanes and alkenes joined by ether linkages^8^. Elongation or condensation of a C_18_ fatty acid with a similar fatty acid molecule may produce C_28_~C_34_ algaenan constituents, which may be catalysed by the action of polyketide synthase^8^. Future work will characterise algaenan composition, biosynthesis, cross-linking, and the role in determining cell wall strength under different nutrient regimens. *N. gaditana* contains seven cellulases in addition to *Upp* and *CesAs* ^8^. Thus, *N. salina* seems to possess the metabolic machinery for cellulose synthesis and degradation. Future work will determine whether these homologs are downregulated under N−, which in turn contributes to cell wall thickening. Characterisation of *N. salina* cellulase homolog(s) would help to define its role in TAG biosynthesis, and may help in exploiting TAG biosynthesis for controlling carbon partitioning to cell wall biosynthesis.

In summary, we demonstrated transcriptional regulation of cellulose biosynthesis in *N. salina* under mild N− conditions, where TAG synthesis is dependent primarily on the supply of photoassimilates from photosynthesis rather than recycling of glycerolipids and the storage polysaccharide laminarin. Under this condition, upregulation of cytosolic FAS1-mediated fatty acid synthesis contributed to augmented TAG synthesis. Further studies on photosynthesis-driven cellulose synthesis have implications for genetic engineering and custom-designed cell wall composition and structure, which would enable easy extraction of the accumulated oils in oleaginous microalgae.

## Methods

### Culture conditions

*Nannochloropsis salina* CCMP1776 was obtained from the Culture Collection of Marine Phytoplankton (CCMP, now the National Centre for Marine Algae and Microbiota, Bigelow Laboratory for Ocean Science, East Boothbay, ME, USA). CCMP1776 cells were maintained on modified f/2 medium^52^ containing 30 g l^−1^ sea salts (Instant Ocean, USA) and 15 g l^−1^ agar under fluorescent illumination (50 µmol m^−2^ s^−1^) with a photoperiod of 16 h light / 8 h dark at 22 °C. Cells were cultivated in 250 ml Erlenmeyer flasks containing 100 ml of medium bubbled with 5% (w/w) CO_2_ at 28 °C with orbital shaking (120 rpm) under fluorescent illumination (80 µmol m^−2^ s^−1^). Nitrogen deficiency was induced as follows: cells at the exponential growth phase (4 × 10^8^ cells ml^−1^) were pelleted by centrifugation (3,500 *g* at 28 °C for 10 min) and washed twice in two different growth media either with nitrogen (427.5 mg l^−1^ NaNO_3_; N+) or without nitrogen (0 mg l^−1^ NaNO_3_; N−). Both cultures were diluted to OD_750_ = 0.15, corresponding to 4 × 10^6^ cells ml^−1^. The algal cells were then allowed to grow for 5 days and sampled twice per each day of culture. To block *de novo* transcription, *N. salina* cultures grown in N+ or N− media were treated with 100 µg ml^−1^ actinomycin D for 24 or 48 h. Algal growth was measured spectrophotometrically by determining the daily changes in OD_750_ (Shimadzu UV 1800, Japan). Algal cell numbers were monitored with a Bürker counting chamber (Marienfeld-Superior, Germany) under a light microscope.

### Growth rate determination, pigment content analysis, and chlorophyll fluorescence measurement

Specific growth rates (μ) were calculated according to the equation, μ = ln (N2/N1)/(t2 − t1), where N2 and N1 equal the total numbers of cells ml^−1^ at time points t2 and t1, respectively, and where t2 > t1. Total chlorophyll *a* (Chl *a*) and carotenoids (Cars) were extracted from cells using 100% N,N′-dimethylformamide (DMF) at 4 °C for 24 h under dark conditions^53^. Pigment concentrations were determined spectrophotometrically using specific extinction coefficients^54^. Changes in *in vivo* Chl fluorescence were monitored through Xe-pulse amplitude modulated (PAM) fluorometry (Walz, Germany) using algal cells that were dark-adapted for 20 min before measurement. The F_v_/F_m_ value, which is an indicator for maximum PSII efficiency, was calculated as (F_m_ − F_0_)/F_m_, where F_v_ is the dark-adapted variable fluorescence, F_m_ is the maximum fluorescence, and F_0_ is the dark-adapted fluorescence. The actual quantum yield of PSII photochemistry in light-adapted algal cells was calculated as 1 − F/F_m_′, where F is steady-state fluorescence and F_m_′ is maximal fluorescence under illumination.

### Light, fluorescence, and electron microscopy

Cellulose and neutral lipids were observed by LSM 800 confocal microscopy (Carl Zeiss, Germany) after staining cells with 0.1% (w/v) calcofluor white (CW) in phosphate buffer at pH 6.0^34^ and 0.0025% (w/v) Nile Red (NR)^19^, respectively. Confocal images were acquired and processed using ZEN 2.1 Lite software. CW and NR fluorescence also was quantified using a spectrofluorometer (LS55, PerkinElmer, USA). CW and NR fluorescence was observed using excitation wavelengths at 300 and 488 nm and emission wavelengths of 400–600 and 500–700 nm, respectively. The relative fluorescence of neutral lipids was obtained by subtracting the autofluorescence of algal cells, CW, and NR. Cells were prepared for electron microscopy as follows. Cell pellets were fixed in freshly prepared primary fixative containing 2.5% glutaraldehyde (w/v) in f/2 medium for 1 h at 4 °C. Then, the cells were rinsed three times with f/2 medium for 10 min each, and post-fixed in a 1:1 mixture of 2% OsO_4_ and 3% ferrocyanide (w/v) for 1 h at 4 °C. Dehydration was performed at 4 °C with a graded series of 50%, 60%, 70%, 80%, and 90% ethanol (10 min each), followed by absolute ethanol (three 10 min washes). Then, cell pellets were brought to room temperature and immersed in two changes of propylene oxide for 20 min each, 50% Spurr’s embedding resin in propylene oxide for 1 h, 75% resin in propylene oxide for 1 h, and then 100% resin overnight. The following day, cell pellets were immersed in fresh 100% resin and polymerised at 70 °C. Polymerised blocks were sectioned into thin layers of 70 nm. Serial sections were collected on formvar-coated slot copper grids, stained with 3% (w/v) uranyl acetate and Reynold’s lead citrate^55^, and then examined and photographed with a JEM-1010 transmission electron microscope operated at 80 kV (JEOL, Tokyo, Japan). Images were recorded on Kodak EM Film 4489 (Eastman Kodak Co., Rochester, NY) and scanned to tagged image file (TIF) format using an Epson Perfection V700 Photo scanner (Epson Korea Co., Ltd., Seoul, Korea). Cell wall thickness was analysed using ImageJ; 250 individual cell walls were measured in five separate places^11^.

### Cell wall extraction and cellulose quantification

Cells were harvested by centrifugation at 3,500 *g* for 10 min, washed three times, and then resuspended in deionised water (dH_2_O). After lyophilisation, whole cells (5 mg DW) were suspended in 0.5 ml dH_2_O and then subjected to bead-beating with a bead beater (bead diameter: 0.1 mm; BioSpec Products, USA) at high speed (1,800 rpm) for 2 min on ice. After centrifugation at 10,000 *g* for 10 min at 4 °C, the cell lysate was washed twice with hot phenol (70 °C) and then three times with chloroform to remove proteins and lipids, respectively. Three cycles of bead-beating were required for almost complete cell lysis. Cell wall extracts were further washed three times in dH_2_O. The laminarin that was pelleted with the cell wall extract was removed by using β-1,3-glucanase (Sigma Cat. No. 67138) treatment at 37 °C for 36 hr. The colour of the purified cell wall was white. Light microscopy revealed that the cell wall extracts isolated in the present study were morphologically identical to purified cell walls. After washing twice in dH_2_O, purified cell wall precipitates were lyophilized and then treated with 1% (w/w) cellulase (Sigma Cat. No. C1184) isolated from *Aspergilus nige*r, which does not exhibit laminarin hydrolysing activity^56^ for 24 h. After centrifugation at 3,500 *g* for 10 min, 10 μl supernatant was mixed with 100 μl Glucose (HK) Assay Reagent (Sigma Cat. No. G3293) and incubated at 25 °C for 15 min. Absorbance at 340 nm was recorded after addition of 100 μl H_2_SO_4_. Glucose was used as a standard to quantify the total cellulose content.

### Analysis of fatty acid methyl esters (FAMEs) by gas chromatography with flame ionisation detector (GC-FID

Total lipids were extracted from 20 mg of freeze-dried samples and transesterified with pentadecane as an internal standard as described previously^57^. The samples were saponified with 2 ml of 7.5 M NaOH:CH_3_OH (1:1, v/v) at 100 °C for 30 min. FAMEs were produced with 4 ml of CH_3_OH:6 N HCl (1:1, v/v) at 80 °C for 10 min, extracted in 2.5 ml of hexane:methyl tetra-butyl ether (1:1, v/v) with shaking for 10 min, and washed with 6 ml of 0.5 M NaOH. The extracted FAMEs were analysed by gas chromatography (YL-6100GC; YoungLin Science, Anyang, Korea) equipped with a FID and an INNOWAX capillary column (30 m × 0.32 mm × 0.5 μm; Agilent Technologies, Santa Clara, CA, USA). Each FAME component was identified and quantified using the Supelco^®^37 Component Fatty Acid Methyl Ester Mix (Sigma). Methyl tridecanoate (C1_3_Me) was used as the recovery standard.

### RNA extraction, cDNA synthesis, and quantitative real-time PCR (qRT-PCR) analysis

Cells were harvested at the mid-exponential phase. Then, total RNA was extracted from 200 mg of wet biomass using NucleoZol (Macherey-Nagel, Germany), and DNA was removed by incubating the extracts with DNaseI (Macherey-Nagel, Germany). The integrity of the purified total RNA was assessed by electrophoresis on 1.2% agarose gels. Then, cDNA was synthesised from total RNA using the iScript cDNA Synthesis kit (Bio-Rad, USA), and qRT-PCR analysis was performed using the CFX96 Real-Time system (Bio-Rad, USA). Primers for PCR reactions were designed to analyse the expression levels of selected key genes in the cellulose and lipid biosynthesis pathways (Supplementary Table S2). The housekeeping gene ubiquitin was used as the internal standard. PCR reactions contained 4 μl of cDNA (for 10 ng of total RNA), 1 μl each of 10 pM forward and reverse primers, 4 μl of deionised water, and 10 μl of SYBR Green Supermix (Bio-Rad, USA) in 20 μl total volume. The PCR reactions were incubated at 95 °C for 3 min, followed by 40 cycles of 95 °C for 10 s, 58 °C for 30 s and 72 °C for 30 s, and then 95 °C for 10 s, followed by a final melting step at 65−95 °C for 5 min. Total RNA and cDNA quantities were measured using the NanoDrop spectrophotometer. Gene expression was calculated using the 2^−∆∆Ct^ method and the CFX Manager program (Bio-Rad). Statistical significance was assessed with Student’s *t*-test. The absolute numbers of transcripts (transcript copies/pg of cDNA) in the presence of actinomycin D were quantified by the cDNA-based absolute qPCR method, where transcript copy number is given by the following equation: (Avogadro’s constant × transcript quantity)/MW^58^.

### Cell breakage and viability assays

Two methods were used for cell disruption. Cells were subjected to sonication with a sonicator (Sonic and Materials, USA) at a resonance of 40 W, two times with 5 s operation, with 5 s intervals. Cells also were subjected to bead-beating with a bead beater (bead diameter: 0.1 mm; BioSpec Products, USA) at high speed (1,800 rpm) for 15 s on ice. After cell disruption by sonication or bead-beating, cells were plated on fresh f/2 growth medium supplemented with 1.5% agar and incubated at 22 °C. Colonies were counted after 25 days. The efficiency of cell disruption was expressed as the cell viability (estimated by cell numbers) before and after disruption.

### Statistical analysis

Data were analysed by two-tailed *t*-tests after normality assessment; *p*-values less than 0.05 were considered statistically significant.

## Electronic supplementary material

Supplemental information
