# Supplementary material for: Transcriptional Regulation of Cellulose Biosynthesis during the Early Phase of Nitrogen Deprivation in Nannochloropsis salina
Source: Sci Rep. 2017 Jul 13;7:5264. doi: 10.1038/s41598-017-05684-4 (PMC5509672; doi:10.1038/s41598-017-05684-4)
Supplement: Supplementary file 1 — Supplemental information [file 41598_2017_5684_MOESM1_ESM.pdf]

Supplementary Materials

Transcriptional Regulation of Cellulose Biosynthesis during the Early Phase of Nitrogen Deprivation in *Nannochloropsis salina*

Seok Won Jeong[1], Seung Won Nam[2], Kwon HwangBo[3], Won Joong Jeong[3], Byeong-ryool Jeong[4], Yong Keun Chang[4,5] & Youn-Il Park[1, *]

[1]Department of Biological Sciences, Chungnam National University, Daejeon 34134, Korea
[2]Bioresources Culture Collection Division, Nakdonggang National Institute of Biological Resources, Sangju 37242, Korea
[3]Korea Research Institute of Bioscience and Biotechnology, Daejeon 34141, Korea
[4]Department of Chemical and Biomolecular Engineering, Korea Advanced Institute of Science and Technology, Daejeon 34141, Korea
[5]Advanced Biomass R&D Center (ABC), Korea Advanced Institute of Science and Technology, Daejeon 34141, Korea

[*]Corresponding author: Youn-Il Park
E-mail: yipark@cnu.ac.kr; Phone: +82-42-8215493; FAX: +82-42-8229690

Supplementary Figure Legends

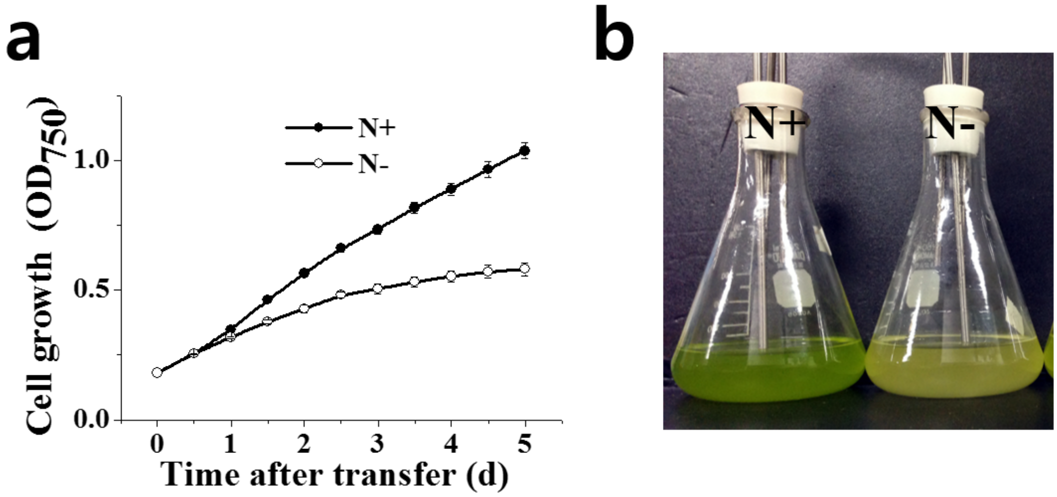

Figure S1. Cell growth (a) and culture images (b) of *N. salina* cells grown under N+ and N– conditions for 2 days. Each data point represents the mean ± SE of three biological and technical triplicate culture flasks.

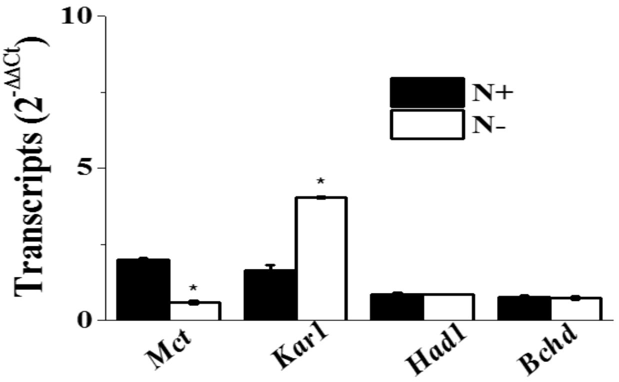

Figure S2. Transcript levels of chloroplast fatty acid synthesis genes in *N. salina* cells grown under N+ and N– conditions for 2 days. Each data point represents the mean ± SE of three biological and technical triplicate culture flasks.

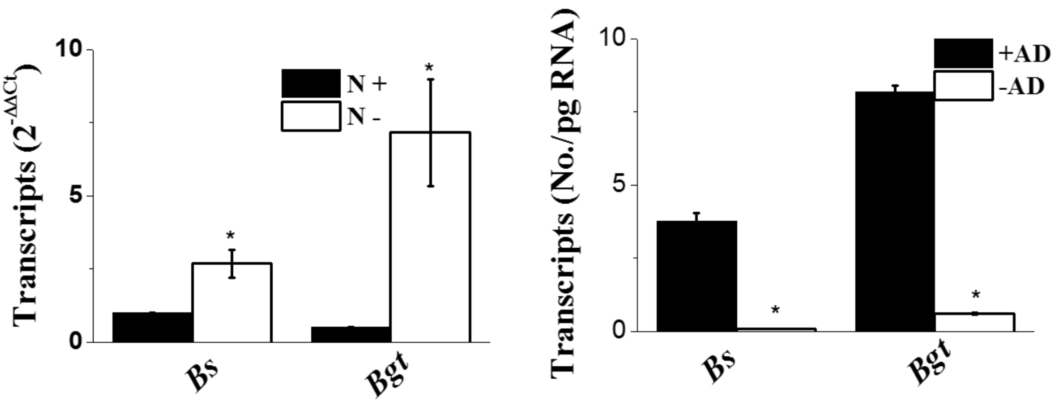

Figure S3. Transcript levels of laminarin (*Bs* and *Bgt*) synthesis genes in *N. salina* cells. Cells were under N+ and N– conditions for 2 days. The transcription inhibitor actinomycin D (AD, 100 µg ml^{-1}) was included in N– media for 2 days before the onset of N– growth. Absolute copy numbers of the mRNA transcripts (number of copies/ng of total RNA) were determined by qRT-PCR analysis. Each data point represents the mean ± SE of three biological and technical triplicate culture flasks.

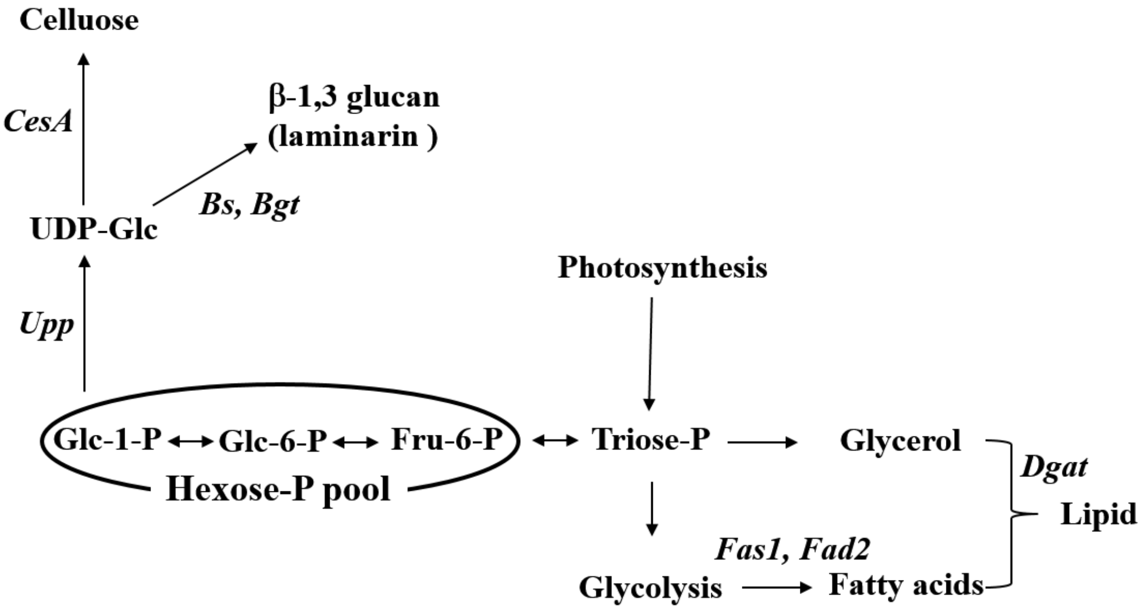

Figure S4. Carbon partitioning in N-deprived *N. salina*. Under N-deficient conditions, total fatty acid and neutral lipid contents increase due to conversion of photosynthate assimilates via transcriptional activation of putative cytoplasmic fatty acid synthase (*Fas1*), Δ12-desaturase (*Fad2*), and diglyceride acyltransferase (*Dgat*). Partitioning of hexose phosphate pools into cellulose biosynthesis appears to be transcriptionally regulated by the upregulation of UDP-glucose (Glc) pyrophosphorylase (*Upp*) and cellulose synthase (*CesA*) activities. N-deficiency increases laminarin biosynthesis in *N. oceanica*. Repression of 1,3-β-glucan synthase (*Bs*) and β-1,3-glucosyltransferase (*Bgt*) transcript levels by the transcription inhibitor actinomycin D in *N. salina* indicated the concerted regulation of photoassimilate partitioning into storage and structural carbohydrates in response to N status.

Supplementary Table S1. Effect of the transcription inhibitor actinomycin D on neutral lipid and cellulose content in *N. salina* cells under nitrogen-depleted (N–) conditions. Neutral lipid and cellulose contents were estimated by Nile Red fluorescence amplitude at 580 nm (F_{580}) and calcofluor white amplitude at 420 nm (F_{420}) relative to those before N– induction, respectively. Data are expressed as the average of three biological replicates ± SD. Each sample was significantly different from the other (independent two-tailed *t*-test, *p*-value <0.05).

	Neutral lipid (F_{580})	Cellulose (F_{420})
N–	12.18 ± 0.67	1.50 ± 0.08
N– + actinomycin D	9.48 ± 0.68	0.79 ± 0.07

Supplementary Table S2. Primer sequences for qRT-PCR analysis.

Gene	Description	Primer forward (5′ to 3′)	Primer reverse (5′ to 3′)	Reference (GenBank)
Bgt	β-1,3-glucosyltransferase	ATGTCTTGGAGGCCATGGAG	TTTGCTCGTGGTCGTCAGC	AFGQ01002665.1
Bs	1,3-β-glucan synthase	GGCGTGGTAATAAGCGAC	GTTCAGTAAAGCTGATGCC	AFGQ01002722.1
Bcdh	Branched-chain α-keto acid dehydrogenase subunit E2	CTGATCACGCCCACCCTC	CGTTTCTCCTGCGCTTTC	AFGQ01001567.1
CesA8	Cellulose synthase	ATTCAACACTTGTTTAGC	CGGTAGCTGATATTCATG	AFGQ01004415.1
CesA122-1	Cellulose synthase	CCCAAGCTGTATTTGATG	GAGAGGATGTAGTAGAGC	AFGQ01001844.1
Dgat-2	Diacylglycerol acyltransferase type 2-5	GATGATGAGCTGGTACTACC	GCTTCCTCCAGAATGTATTCG	AFGQ01000682.1
Fad2	Δ12 desaturase	CACCCTTGCTGACATCAAAG	CGCATTCATGGGCAATCAC	AFGQ01000627.1
Fad1	Δ5 desaturase	CTACATGCTGCGAGACTTTG	GGTTCTCGAGCAAGGCTTG	AFGQ01001226.1
Fas1a	Type I fatty acid synthase	ACACGGCGTGCTCGTCATC	GCATCAAGCATAAGATTCAC	KK037387.1
Fas1b	Type I fatty acid synthase	TTGCAGCGTGTAGTGCTG	AAGCATCTCCCAAAAAGCC	KK037387.1
Had1	3-hydroxy acyl ACP dehydratase	ATGGAGGTGGAACTGGTC	CTTGATTTCGACGGCCAC	AFGQ01000652.1
Kar1	3-ketoacyl-ACP reductase 1	GACGGCATTAAGAAGGCG	TAGCAATGCCTCCATCGAC	AFGQ01000162.1
Mct	Malonyl-CoA:ACP transacylase	CGAATGACAGTGCGTCTG	GAACTCTGTCTTGGCCAAC	AFGQ01000324.1
Pksa	Type I fatty acid synthase-like	CTTGAGGTTGGGTATACTGC	GAATATACCAACGTTTCGCG	KK037387.1
Pksb	Type I fatty acid synthase-like	ACTGCTTTTGTTGCCCTCGC	GAATGACTTGCATTTACCATC	AFGQ01003363.1
Pksc	Type I fatty acid synthase-like	CCGAGTATATTTTCGACGAC	ATATTGCACGGCAGCAAGGC	AFGQ01000753.1
Pkse	Type I fatty acid synthase-like	GATCGAACGCAGTCTTCG	TTGAATCTTTGTCTCCTTGC	AFGQ01000951.1
Ubq	Ubiquitin	GGCAAGACGATCACACTGGA	AAAGCGCGTCTCCACCAC	AFGQ01000799.1
Upp	UDP-glucose pyrophosphorylase	CGCATACCAAATGGGGTC	TTTAGCGTCACATTCGCG	AFGQ01003838.1
